# Temporomandibular disorders in an adult population in northern Norway: A cross‐sectional study

**DOI:** 10.1002/cre2.463

**Published:** 2021-06-16

**Authors:** Elin Hadler‐Olsen, Elizabeth Thon, Gro Eirin Holde, Birgitta Jönsson, Nils Oscarson, Anders Tillberg

**Affiliations:** ^1^ The Public Dental Health Service Competence Centre of Northern Norway Tromsø Norway; ^2^ Department of Medical Biology, Faculty of Health Sciences UiT the Arctic University of Norway Tromsø Norway; ^3^ The Public Dental Health Service Tromsø Norway; ^4^ Department of Clinical Dentistry, Faculty of Health Sciences UiT the Arctic University of Norway Tromsø Norway; ^5^ Department of Periodontology, Institute of Odontology The Sahlgrenska Academy at the University of Gothenburg Sweden

**Keywords:** dysfunction, epidemiology, oral health survey, quality of life, temporomandibular joint disorder

## Abstract

**Objectives:**

The aim of the study was to assess the prevalence of symptoms indicative of temporomandibular disorders (TMD) in an adult population in Troms County in Northern Norway, as well as the associations between TMD and socio‐demographic factors, dental status, self‐reported general, and oral health as well as oral health related quality of life (OHQoL).

**Methods:**

Data were collected from a structured questionnaire and a clinical examination of a random sample of almost 2000 adults, 20–79‐year‐old, in Troms County in Northern Norway.

**Results:**

Women had a higher prevalence of all self‐reported and clinical signs of pain and dysfunction in the temporomandibular complex compared to men. For both genders, sounds from the temporomandibular joint (TMJ) upon clinical examination was the most common symptom, followed by pain to palpation of jaw muscles. Headache was the most common of the self‐reported symptoms and sounds from the TMJ the second most common. Young women had a higher prevalence of self‐reported headache and jaw‐ and face pain compared to middle‐aged and elderly women. TMD‐related symptoms of pain were significantly associated with poor self‐reported general health and correlated with OHQoL as assessed by the oral health impact profile 14 questionnaire.

**Conclusion:**

Being women and having moderate to poor self‐reported general health were associated with clinical signs and self‐reported symptoms of pain in the jaw, face and head region. Self‐reported symptoms of TMD correlated more strongly with OHQoL than clinical signs.

## 1 INTRODUCTION

Pain in the temporomandibular region is a common chronic pain condition, and temporomandibular disorders (TMD) is a collective term used to describe pain and functional disturbances of the masticatory system (List & Jensen, 2017; Lovgren, Haggman‐Henrikson, et al., 2016). Symptoms of TMD include pain in the masticatory muscles or in the jaw, as well as headache in the temple region. TMD also includes symptoms in the temporomandibular joint (TMJ) such as joint sounds and restricted jaw mobility, as well as degenerative joint diseases (Ohrbach & Dworkin, 2016).

Many epidemiological studies have assessed prevalence and symptoms of TMD (Anastassaki et al., 2012; Lovgren, Haggman‐Henrikson, et al., 2016). In a review of six population‐based studies, 6–15% of adult women and 3–10% of adult men had self‐reported pain from the TMJ region (LeResche, 1997). Across cross‐sectional studies, TMD symptoms, for example, pain, are reported to be 1.5–2 times more common in women than in men (LeResche, 1997). TMD symptoms have also been shown to be more common in adolescents and middle‐aged adults than in children and elderly persons (Carlsson et al., 2014; Horswell & Sheikh, 2018; Lovgren, Haggman‐Henrikson, et al., 2016).

For a long time, occlusal disorders were considered the most important risk factor for TMD. However, recent research has shown that the ethology of TMD is multifactorial, and occlusal factors seem to be of minor importance (Carlsson, 2010). Through the prospective, longitudinal OPPERA studies (Orofacial Pain: Prospective Evaluation and Risk Assessment), numerous risk factors have been validated and ranked based on their impact. According to these studies, other health conditions, both painful, and non‐painful ones, are among the most important risk factors for TMD development (Slade et al., 2016). Other prominent risk factors include a high number of unspecific oral symptoms and oral parafunctions such as bruxism, TMJ sounds and reduced mobility of the TMJ. Sociodemographic factors, including age and marital status, variables related to pain sensitivity as well as psychosocial factors are also verified as risk factors of TMD, but of less importance (Ohrbach et al., 2013; Slade et al., 2016).

The clinical relevance of chronic pain conditions may to some extent be evaluated by their effect on the quality of life. Oral conditions are often assessed for their impact specifically on oral health‐related quality of life (OHRQoL) which may be measured through questionnaires such as the Oral Impact on Daily Performance (OHIP). There are few large studies assessing the association between symptoms of TMD and OHRQoL in an adult population (Dahlstrom & Carlsson, 2010; Oghli et al., 2020). Furthermore, there are no previous studies reporting prevalence of symptoms related to TMD and their association to OHRQoL in a Norwegian adult population. Studies from other Nordic countries, including Sweden and Finland exists, but despite many similarities between Nordic countries, there are work‐related differences, such as employment‐rate, average working hours and sickness benefits between the counties (https://www.nordicstatistics.org/), as well as cultural and life‐style differences that may affect disease burden (Nordic Burden of Disease C, 2019). It is therefore important to provide TMD data for a Norwegian population. The cross‐sectional, population‐based study called Tromstannen – Oral Health in Northern Norway (TOHNN) assessed both subjective symptoms as well as clinical signs of TMD in almost 2000 adults in Troms County in Northern Norway. The study aimed at assessing the prevalence of subjective and clinical signs indicative of TMD, as well as their association with socio‐demographic factors, general and oral health as well as OHRQoL.

## 2 METHODS

### 2.1 Study design

The data in this project were collected as a part of the TOHNN‐study, a cross‐sectional, population‐based study in Northern Norway that included a structured questionnaire as well as a clinical examination. The sample size calculations, selection, and invitation procedures, as well as calibration routines and attendance rate have been described in detail previously (Holde et al., 2016). In brief, the study was conducted in the years 2013–14, in Troms County, in Northern Norway, and 2.909 adults between 20 and 79 years were invited to participate in the study. From the original study sample 1.986 people participated of whom 1.946 (66.8% of the invited) had a complete questionnaire as well as data from the clinical examination of the temporomandibular complex. The regional Committee for Medical and Health Research Ethics of Northern Norway, Norway, approved the study (2013/348/REK Nord). All participants provided a written informed consent.

### 2.2 Variables from the questionnaire

Participants completed a questionnaire containing 49 questions assessing socio‐demographic conditions, general health, oral health and self‐reported dysfunctions, and pain in the face/head region. The independent variables used in the present study and their distribution are listed in Table 1, and they were chosen based on previous reports of their association to TMD.

**TABLE 1:** Background data. Valid number and per cent are presented in each row by gender

	Women	Men		Total
	*N* (%)	*N* (%)	*p‐*value^#^	*N* (%)
Gender	996 (51.2)	950 (48.8)		1946 (100)
Age groups				
20–39	344 (34.5)	268 (28.2)		612 (31.4)
40–59	400 (40.2)	405 (42.6)		805 (41.4)
60+^*^	252 (25.3)	277 (29.2)	<0.01	529 (27.2)
Municipality				
Rural^*^ (< 10,000)	222 (22.3)	253 (26.6)		475 (24.4)
Sub‐urban (10,000–50,000	302 (30.3)	300 (31.6)		602 (30.9)
Urban (> 50,000)	472 (47.4)	397 (41.8)	<0.05	869 (44.7)
Household income				
<450,000	314 (33.2)	279 (29.1)		584 (31.2)
450,000–899,999	450 (47.6)	472 (51.0)		922 (49.3)
≥900,000^*^	182 (19.2)	184 (19.9)	n.s.	366 (19.5)
Education				
Secondary school^*^	154 (15.6)	149 (15.8)		303 (15.7)
High school	391 (39.6)	442 (47.1)		833 (43.3)
University level	443 (44.8)	348 (37.1)	<0.01	791 (41.0)
Self‐reported general health				
Good^*^	717 (72.3)	692 (73,0)		1409 (72.6)
Moderate	231 (23.3)	222 (23.4)		453 (23.4)
Poor	44 (4.4)	34 (3.6)	n.s.	78 (4.0)
Use of prescribed medicine				
No^*^	544 (54.9)	552 (58.7)		1096 (56.7)
Yes	447 (45,1)	389 (41.3)	n.s.	836 (43.3)
Smoking habits				
Non‐smoker^*^	833 (84.0)	808 (85.7)		164 (84.9)
Current smoker	159 (16.0)	134 (14.3)	n.s.	293 (15.1)
Self‐reported oral health				
Good^*^	527 (53.5)	404 (42.7)		931 (48.2)
Moderate	352 (35.7)	389 (41.1)		741 (38.4)
Poor	106 (10.8)	153 (16.2)	<0.01	259 (13.4)

^#^ *p*‐value for differences between groups using the χ^2^ test.

*Note*:n.s. = not significant.

^*^ The reference group for the related independent variables in the regression analysis is represented with an asterix (*).

Participants reported their birth year in the questionnaire, and for statistical analyses age was stratified into three groups, 20–39 years, 40–59 years, and ≥60 years, based on former age‐stratified prevalence data reporting a significant decrease in TMD symptoms in women after the age of 40, and in men after the age of 60 (Lovgren, Haggman‐Henrikson, et al., 2016). General and oral health was rated by the participants on a 5‐graded scale, and answers were categorized as good (very good, fairly good), moderate (neither good nor poor), and poor (fairly poor, very poor). The impact of OHRQoL was assessed by the Oral Health Impact Profile (OHIP) 14 questionnaire (Slade, 1997). By 14 questions, this assesses how often during the past year the respondent has experienced negative impact from mouth, teeth or dentures on various aspects of oral function, social and psychological well‐being and pain. The frequency is reported on a 5‐graded scale from never (0) to often (4). The oral health impact was assessed using the sum‐score of all questions with a possible range from 0 to 56. Missing values for the individual parameters were replaced with the median, and if answers were missing for more than one parameter, the respondent was excluded.

The questionnaire included two questions assessing symptoms of TMD related pain, namely headache or pain from the jaw or face, as well as three questions assessing symptoms of dysfunction including TMJ locking, TMJ sounds and difficulty opening the mouth wide. These variables are referred to as “self‐reported.” The frequency of each symptom was assessed on a 5‐graded scale, and subsequently dichotomised into occurring once a week or more and occurring less frequently. The registration and categorization of general and oral health, self‐reported dysfunction, and self‐reported pain were done as described in a similar study to allow comparison.

A self‐reported TMD pain variable based on the question “pain in jaw‐face region” and “headache,” as well as a self‐reported TMD dysfunction variable based on the questions “difficulty opening the mouth wide,” “TMJ sounds,” and “jaw locking” were created. They were dichotomous with cut‐off between symptoms occurring at least once a week, and more seldom, and they were used as outcome variables in regression analysis (Table 3). The questions assessing occurrence of pain once a week or more in the facial region have been validated and found to have high sensitivity and high specificity with reference to TMD, and the frequency cut‐off at once a week or more increased the reliability (Lovgren, Haggman‐Henrikson, et al., 2016; Nilsson et al., 2006; Wahlund et al., 1998).

### 2.3 Clinical examination

The clinical examination of the temporomandibular complex was based on previously described protocols and included function of the TMJ and pain to palpation. Variables based on the clinical examination are referred to as “clinical signs.” To register TMJ clicking or crepitation, the examiner placed their index fingers on the TMJ and registered sounds during three jaw opening‐closing cycles. TMJ pain was assessed by applying a light pressure with the index fingers over the TMJ, and the patient indicted if this provoked tenderness or pain. TMJ pain during unassisted jaw movement was also registered. Jaw muscle pain to palpation was assessed by bilateral palpation with the index fingers of the anterior and posterior parts of the temporal muscles, the origin and body of the masseter muscles and the lateral pterygoid muscles' origins. TMJ pain and jaw muscle pain were registered as “present” or “not present.”

Maximum unassisted opening capacity was measured by a ruler, and the vertical over‐jet between 11 and 41 during occlusion in IP was added to the largest distance between the incisal edges of 11 and 41 on maximum opening. If maximum mouth opening provoked pain, maximum opening without pain was also measured. To assure a common understanding of definitions and to calibrate the clinical examiners, one of the authors (AT) examined two persons together with each of the 11 teams involved in the study prior to start. During the study period, AT also visited the four clinics conducting the study and repeated the calibration procedures and discussed diagnoses with each of the examiner teams. As the examiner teams were spread out in a large geographical area, it was not doable for all the examiners to inspect the same participants, thus no inter‐observer correlation was calculated.

Two dichotomous outcome variables, “Clinical signs of TMD pain” (“jaw muscle pain to palpation,” “TMJ pain to palpation,” and “pain during jaw movements”) and “Clinical signs of TMD dysfunction” (“TMJ sounds” and “jaw opening of less than 40 mm”), were created for regression analysis.

## 3 STATISTICAL ANALYSES

All statistical analyses were performed using the IBM® SPSS® statistics 25 for Mac personal computer. Chi‐square test was used to analyze differences between groups. A Post Hoc analyses were made by using Bonferroni Chi Square Residual Analysis (Table 2). Logistic regression analysis was performed with dependent and independent variables listed in Tables 3 and 4. The independent variables that showed significant association with the dependent variables in univariate regression analysis were included in a multivariate model. The results are presented as odds ratios (OR) with 95% confidence intervals (95% CI). Since basic regression texts highlight that variables non‐significant at the univariate level can become significant at the multivariate level of analysis, we also made regression analyses with all variables involved in the analyses. No significant changes were registered.

**TABLE 2:** Distribution of clinical signs^a^ and self‐reported^b^ symptoms stratified by gender and age group. Valid number and per cent. (Test for difference between age groups was made by chi 2 analysis. A post hoc analyses were made by using Bonferroni Chi Square residual analysis)

	Women (*n* = 996)							Men (*n* = 950)						
Age group	20–39 (*n* = 344)		40–59 (*n* = 400)		60+ (*n* = 252)			20–39 (*n* = 268)		40–59 (*n* = 405)		60+ (*n* = 277)		
	*N*	%	*N*	%	*N*	%	*p*‐value	*N*	%	*N*	%	*n*	%	*p*‐value
Clinical signs														
*Pain*														
TMJ pain	61	17.7	71	17.8	38	15.1	n.s.	22	8.2	30	7.4	16	5.8	n.s.
Jaw muscle pain	79	23.0	82	20.5	53	21.0	n.s.	19	7.1	30	7.4	18	6.5	n.s.
Pain open mouth	36	10.5	38	9.5	14	5.6	n.s.	11	4.1	12	3.0	5	1.8	n.s.
*Dysfunction*														
TMJ sounds	131	38.1	167	41.8	107	42.4	n.s.	56	20.9	93	22.9	95^*^	34.3	<0.01
Max open. Cap. below 40 mm	12	3.5	14	3.5	19*	7.5	<0.05	3	1.1	7	1.7	12^*^	4.3	<0.05
Self‐reported symptoms														
*Pain*														
Jaw‐face pain	27	7.8	22	5.5	9	3.6	n.s.	3	1.1	12	3.0	5	1.8	n.s.
Headache	63^*^	18.3	53	13.3	19	7.5	<0.01	16	6.0	23	5.7	9	3.2	n.s.
*Dysfunction*														
Difficult open mouth wide	13	3.8	12	3.0	6	2.4	n.s.	4	1.5	5	1.2	6	2.2	n.s.
TMJ sounds	53^*^	15.4	44	11.0	20	7.9	<0.05	26^*^	9.7	12	3.0	16	5.8	<0.01
TMJ locking	3	0.9	3	0.8	3	1.2	n.s.	0	0.0	1	0.2	1	0.4	n.s.

^*^ Cells where a significant *p*‐value appears.

^a^ Based on clinical examination.

^b^ Based on questionnaire.

**TABLE 3:** Univariate and multivariate models present a domain of clinical signs of pain in the jaw‐face‐region and a domain of self‐reported symptoms of pain in the temporomandibular joint

		Clinical pain		Self‐reported pain	
Variables	*N* (%)	Univariate OR (95% CI)	Multivariate OR (95% CI)	Univariate OR (95% CI)	Multivariate OR (95% CI)
Gender					
Men	950 (48.8)	REF	REF	REF	REF
Women	996 (51.2)	2.8 (2.2–3.5)^***^	2.9 (2.3–3.6)^***^	2.7 (2.0–3.7)^***^	2.8 (2.0–3.8)^***^
Age group					
20–39	612 (31.4)	1.2 (0.9–1.5)	1.3 (1.1–1.8)^**^	1.7 (1.2–2.6)^***^	2.3 (1.5–3.5)^***^
40–59	805 (41.4)	1.5 (1.1–1.9)^**^	1.8 (1.3–2.5)^*^	2.4 (1.6–3.5)^***^	4.3 (2.7–6.9)^***^
60+	529 (27.2)	REF	REF	REF	REF
Municipality					
Rural	475 (24.4)	REF		REF	
Sub‐urban	602 (30.9)	0.9 (0.7–1.2)		1.3 (0.9–1.9)	
Urban	869 (44.7)	0.7 (0.5–1.0)		1.5 (1.0–2.2)	
Household income					
<450,000	584 (31.2)	1.2 (0.9–1.7)		2.0 (1.3–3.2)^**^	
450,000 – 899,999	922 (49.3)	1.4 (1.0–1.9)		1.8 (1.2–2.9)^**^	
**>900,000**	366 (19.5)	REF		REF	
**Education**					
Secondary school	303 (15.7)	REF		REF	
High school	833 (43.3)	0.7 (0.5–1.0)		0.8 (0.5–1.2)	
University level	791 (41.0)	0.8 (0.6–1.1)		0.7 (0.5–1.1)	
Self‐reported general health					
Good	1409 (72.6)	REF	REF	REF	REF
Moderate	453 (23.4)	1.5 (1.2–1.9)^*^	1.4 (1.1–1.9)^*^	2.5 (1.8–3.4)^***^	2.3 (1.6–3.3)^***^
Poor	78 (4.0)	3.1 (1.9–4.7)^***^	2.6 (1.6–4.4)^***^	5.3 (3.2–8.9)^**^	4.2 (2.3–7.6)^***^
Use of prescribed medication					
No	1096 (56.7)	REF		REF	REF
Yes	836 (43.3)	1.3 (1.1–1.7)^**^		2.0 (1.5–2.7)^**^	2.6 (1.9–3.6)^**^
Smoking habits					
Non‐smoker	1641 (84.9)	REF		REF	
Current smoker	293 (15.1)	1.4 (1.1–1.9)^*^		0.6 (0.4–0.8)^**^	
Self‐reported oral health					
Good	931 (48.2)	REF	REF	REF	REF
Moderate	741 (38.4)	1.1 (0.9–1.4)	1.3 (0.9–1.5)	1.6 (1.2–2.2)^**^	1.7 (1.2–2.4)^***^
Poor	259 (13.4)	1.7 (1.3–2.3)^***^	1.7 (1.2–2.4)^**^	2.5 (2.7–3.7) ^***^	2.8 (1.8–4.4) ^***^

* *p*<0.005; ** *p*<0.01; *** *p*<0.001.

**TABLE 4:** Univariate and multivariate models present a domain of clinical signs of dysfunction in the jaw‐face‐region and a domain of self‐reported symptoms of dysfunction in the temporomandibular joint

		Clinical dysfunction		Self‐reported dysfunction	
Variables	*N* (%)	Univariate OR (95% CI)	Multivariate OR (95% CI)	Univariate OR (95% CI)	Multivariate OR (95% CI)
Gender					
Men	950 (48.8)	REF	REF	REF	REF
Women	996 (51.2)	2.0 (1.6–2.4)^***^	2.0 (1.7–2.4)^***^	2.0 (1.5–2.8)^***^	1.9 (1.4–2.7)^***^
Age group					
20–39	612 (31.4)	0.7 (0.5–0.8)^*^		1.0 (0.6–1.5)	0.9 (0.6–1.4)
40–59	805 (41.4)	0.7 (0.5–0.9)^**^		1.8 (1.3–2.8)^**^	1.8 (1.2–2.6)^**^
60+	529 (27.2)	REF		REF	REF
Municipality					
Rural	475 (24.4)	REF	REF	REF	
Sub‐urban	602 (30.9)	1.4 (1.1–1.7)^*^	1.5 (1.2–1.9)^*^	1.2 (0.8–1.9)	
Urban	869 (44.7)	0.8 (0.6–1.1)	0.8 (0.6–1.1)	1.4 (1.0–2.3)	
Household income					
<450,000	584 (31.2)	1.2 (0.9–1.6)		1.4 (0.9–2.2)	
450,000–899,999	922 (49.3)	1.3 (1.0–1.7)		1.4 (0.9–2.3)	
>900,000	366 (19.5)	REF		REF	
Education					
Secondary school	303 (15.7)	REF		REF	
High school	833 (43.3)	0.7 (0.5–0.9)^*^		1.1 (0.7–1.8)	
University level	791 (41.0)	0.8 (0.6–1.0)		1.2 (0.7–1.9)	
Self‐reported general health					
Good	1409 (72.6)	REF		REF	
Moderate	453 (23.4)	1.2 (1.0–1.5)		1.4 (1.0–1.9)	
Poor	78 (4.0)	1.2 (0.8–1.9)		1.7 (0.9–3.4)	
Use of prescribed medication					
No	1096 (56.7)	REF	REF	REF	
Yes	836 (43.3)	1.4 (1.2–1.7)^***^	1.3 (1.1–1.6)^*^	1.1 (0.8–1.5)	
Smoking habits					
Non‐smoker	1641 (84.9)	REF		REF	
Current smoker	293 (15.1)	1.2 (0.9–1.6)		0.9 (0.6–1.4)	
Self‐reported oral health					
Good	931 (48.2)	REF		REF	
Moderate	741 (38.4)	1.0 (0.8–1.3)		1.4 (1.0–2.0)	
Poor	259 (13.4)	1.1 (0.8–1.4)		1.5 (0.9–2.3)	

* *p*<0.005; ** *p*<0.01; *** *p*<0.001.

Correlations between OHIP14 sum‐score and the constructed dependent variables were analysed by Spearman's correlation analyses, due to the non‐normal distribution of OHIP14 sum scores. Median values are given with interquartile range (IQR). Differences in OHIP14 sum‐score between groups were analyzed by independent samples median test. For all analyses the significance level was set at 0.05.

Due to variations in missing data, the number of individuals included in analyses varied. An analysis of missing data patterns by the Multiple Imputation method showed that the missing values appeared to be random. The largest numbers of missing data were found in the variable “Household income” (3.8%). Missing values were excluded in the statistical analyses.

## 4 RESULTS

### 4.1 Study cohort

The socio‐demographic and health characteristics of the study cohort are presented in Table 1.

The mean number of teeth was 25.0, and 49 participants (2.5%) were edentulous.

### 4.2 Clinical signs and self‐reported symptoms of pain and dysfunction

The gender and age distribution of clinical signs and self‐reported symptoms of pain and dysfunction in the temporomandibular complex are listed in Table 2, which shows a higher prevalence of clinical signs indicative of TMD compared to self‐reported symptoms across age groups and gender. TMJ sound was the most common clinical sign of TMD, followed by jaw muscle pain to palpation. TMJ‐ and jaw muscle pain to palpation were significantly more prevalent in women than in men (16.9% and 21.5%, respectively, for women and 7.1% and 7.0%, respectively, for men, *p* < 0.05), but with no significant differences between age groups. Although both women and men in the oldest age group most often experienced restricted mouth opening capacity, they did not experience more pain upon mouth opening than the younger age groups (Table 2). Altogether, 30.4% of women and 13.5% of men had one or more clinical sign of TMD pain, and 43.1% of women and 27.5% of men had one or more clinical sign of dysfunction.

Headache was by far the most common self‐reported symptom of pain. Women had headache more often than men, (13.3% and 5.9%, respectively, *p* < 0.05), and it was more common in the young‐ and middle‐aged groups than in the old‐age group. Participants with self‐reported headache more often had clinical signs of TMD such as TMJ sounds (*p* < 0.05), TMJ‐ and jaw muscles pain to palpation and pain when opening the mouth wide (*p* < 0.001). Jaw‐face pain was reported by 5.6% of women – with the highest frequency in the youngest age group, and by 2.0% of men with no significant difference in prevalence between the age groups.

TMJ sound was the most common self‐reported symptoms of dysfunction in both genders, with the highest prevalence among young women (Table 2). Overall, 16.1% of women and 6.5% of men reported one or more symptom of TMD pain, and 12.2% of women and 6.5% of men reported one or more symptom of TMD dysfunction.

Univariate analyses were conducted between variables previously reported to be associated with TMD, and the constructed outcome variables clinical signs pain, self‐reported pain, clinical signs of dysfunction, and self‐reported dysfunction (Tables 3 and 4). Gender, age, and self‐reported general‐ and oral health were significantly associated with both clinical signs and self‐reported symptoms of TMD pain in multivariate analyses (Table 3). Female gender and having poor general health were most strongly associated with clinical signs of TMD associated pain, whereas being middle‐aged and having poor general health were most strongly associated with self‐reported TMD associated pain. Respondents using prescription medication had higher odds of self‐reported symptoms, but not clinical signs of TMD‐associated pain in multivariate analyses (Table 3).

Female gender, living in a suburban municipality and use of prescription medication were associated with clinical symptoms of TMD dysfunction in multivariate analyses (Table 4). Odds ratios for self‐reported symptoms of TMD dysfunction, were higher for woman compared to men and for middle‐aged compared to old‐aged, in both univariate and multivariate analyses (Table 4).

### 4.3 Oral health related impact on daily life

Median OHIP14 score for the whole study population was 3 (IQR = 1–8), with no significant gender differences (median 3 (IQR = 1–7) and 3 (IQR = 1–8) for men and women, respectively, *p* = 0.770). Respondents in the youngest age group had a significantly higher median OHIP14 sum‐score compared to the other respondents (4 (IQR = 1–9) vs. 2 (IQR1‐7) and 3 (IQR = 1–8) in the 20–39 years, 40–59 years, and ≥60 years age groups, respectively, *p* < 0.001). OHIP14 sum‐score was significantly correlated to the four constructed outcome variables: self‐reported pain, self‐reported dysfunction, clinical signs of pain and clinical signs of dysfunction (Table 5). The correlations were stronger for the self‐reported‐ than for the clinical outcome variables, with the strongest correlation with self‐reported pain and the weakest with clinical signs of dysfunction. The OHIP14 sum‐score was more strongly correlated to symptoms of TMD pain and dysfunction in women than in men. Self‐reported pain was most strongly correlated with OHIP14 sum‐score in the youngest age group, otherwise there were no marked differences between age‐groups (Table 5).

**TABLE 5:** Spearman's correlation between Oral health impact profile (OHIP) 14 sum‐score and self‐reported symptoms and clinical signs of TMD pain and dysfunction

*r*_s_	OHIP14 sum‐score					
	All (*N* = 1946)	Men (*N* = 950)	Women (*N* = 996)	20–39 years (*N* = 612)	40–59 years (*N* = 805)	≥60 years (*N* = 529)
Self‐reported pain	0,287^a^	0,281^a^	0,313^a^	0,355^a^	0,228^a^	0,238^a^
Self‐reported dysfunction	0,258^a^	0,230^a^	0,287^a^	0,265^a^	0,200^a^	0,264^a^
Clinical signs of pain	0,175^a^	0,126^a^	0,221^a^	0,120^a^	0,211^a^	0,170^a^
Clinical signs of dysfunction	0,055^b^	0,046	0,067^b^	0,091^b^	0,027	0,081

*Note: r*_s_ = Spearman's rank correlation coefficient.

a . Correlation is significant at the 0.01 level (2‐tailed).

b . Correlation is significant at the 0.05 level (2‐tailed).

### 4.4 Analyses of excluded participants

There were no significant differences between the participants with complete temporomandibular records (*n* = 1.946), compared with those excluded due to incomplete data (*n* = 40) for the variables gender, income and residence. There was a significant difference in mean age between the excluded group and included participants for both genders; 46.8 years versus 49.1 years for men, and 50.6 years versus 46.9 years for women among excluded and included participants, respectively.

## 5 DISCUSSION

The present study is the first to report prevalence of symptoms related to TMD as well as correlation data between TMD and OHRQoL in a Norwegian population. We found that clinical signs of TMD pain and dysfunction were more prevalent than self‐reported symptoms, with TMJ sounds and jaw muscle pain to palpation being the most frequently encountered. The self‐reported symptoms of TMD were more strongly correlated to OHRQoL than the clinical sign. Both clinical signs and self‐reported symptoms were more common in women than in men. TMD associated pain was more common in young and middle‐aged adults compared to older, whereas TMD dysfunction was most common among the elderly. Participants with poor general and oral health had a higher risk of TMD associated pain. Self‐reported pain is often used to report prevalence of TMD in epidemiological studies, and for the present study prevalence of self‐reported jaw‐face pain was 2.0% and 5.6%, for men and women, respectively.

Being a woman significantly increased odd for all the constructed TMD outcome variables in the present study; clinical signs of pain, self‐reported pain, clinical signs of dysfunction and self‐reported dysfunction. This fits well with the gender differences identified in similar cross‐sectional studies and seems to be a consistent pattern regardless of population and study design (Anastassaki et al., 2012; Johansson et al., 2003; Lovgren, Haggman‐Henrikson, et al., 2016). However, the prospective OPPERA studies could not verify a marked gender difference in the incidence of TMD, but rather found that women more often had persistent symptoms of TMD than men did, which may explain the gender differences in prevalence found in cross‐sectional studies (Slade et al., 2016). The gender difference in experience of pain may be related to neuropsychological factors ‐ women have been reported to have a lower pain threshold and report more severe pain, more frequent pain and pain of longer duration than men do (Dao & LeResche, 2000). Women experience variations in hormonal levels throughout life and have a higher prevalence of TMD in puberty, and a reduction after menopause, suggesting that female hormones may have a role in the pathobiology of TMD. The fact that puberty seems to mark the start of the gender difference in prevalence (Lovgren, Haggman‐Henrikson, et al., 2016) also supports this theory. In the present study, the prevalence of TMD‐associated pain among women was highest in the youngest age‐group where the vast majority would be pre‐menopausal, followed by the middle‐aged group where there would be a mix of pre‐menopausal, peri‐menopausal and post‐menopausal women, and lowest in the oldest age‐group where the women would be post‐menopausal. In contrast, men reported no significant difference in prevalence of pain between age‐groups. This aligns well with the theory of female hormones as a modifier of TMD. However, a literature review found only weak and contradicting evidences of a direct role of estrogen in the pathogenesis of TMD (Berger et al., 2015). Estrogen may affect the morphology of the TMJ cartilage and bone, but also modify pain perception, and this spectrum of physiological processes affected by the hormone may cause confusion regarding its precise role in TMD (Berger et al., 2015).

The study population was stratified into three different age groups; young adults (20–39 years), middle‐aged (40–59 years), and old adults (≥60 years) because several previous studies have reported higher prevalence of TMD symptoms in adolescents and middle aged compared to elderly (Lovgren, Haggman‐Henrikson, et al., 2016; Tzakis et al., 1994; Yekkalam & Wanman, 2014a). In line with these studies, we found that the young adults, and most notably the middle aged had a higher risk of having both clinical signs of and self‐reported TMD associated pain compared to those in the oldest age‐group. However, clinical signs of dysfunction were most common in the oldest age‐group. This may partly be due to increased prevalence of osteoarthritis in the elderly, which may give rise to crepitation sounds in the TMJ, in accordance with previous reports (Tzakis et al., 1994). In the present study the older participants also had a higher prevalence of restricted jaw opening than the young and middle‐aged, nevertheless they reported less frequent pain upon mouth opening, and there were no significant differences in self‐reported difficulties in opening the mouth wide between the age‐groups. Although aging is usually associated with a health decline, many studies find that elderly report overall less pain than young and middle‐aged adults. With aging there are functional changes in both the peripheral and central nervous system, including a decreased density of unmyelinated peripheral nerve fibers, and loss of volume in parts of the brain that are associated with pain perception. Furthermore, the experimental pain threshold generally increases with old age, whereas older people may experience pain for longer duration than younger once it begins (Molton & Terrill, 2014). Older people are also found to have a different attitude to pain and more effective coping strategies than younger, which can affect how they report pain. Also, as the general health declines, other symptoms than pain may be more disturbing and affect how they report and deal with both the pain and functional aspects of TMD.

Several studies have found that TMD and self‐reported general health are associated, and the prospective OPPERA studies found that variables related to general health status were among the most significant risk factors for development of TMD, followed by orofacial pain variables (Jussila et al., 2018; Slade et al., 2016). This association was also found in the present study, where poor self‐reported general health was one of the variables that was most strongly associated with TMD related pain. People who reported moderate to poor health were more likely to have both clinical signs of and self‐reported symptoms of TMD pain, as were individuals with moderate to poor self‐reported oral health. A previous study found that other health conditions, for example, headache, depression, sleep apnoea, fibromyalgia, and arthritis, were more than six times more likely to occur after TMD was diagnosed (Hoffmann et al., 2011). The reason for the strong association between TMD and other diseases and pain conditions – both general and oral – are not fully understood. Longitudinal studies have suggested that TMD may be related to a persistent reduction in pain threshold (Slade et al., 2014). Also, chronic TMD have been related to single nucleotide polymorphisms in genes involved in both peripheral and central pain sensation and interpretation, that may have a role in the pathobiology of TMD (Slade et al., 2016).

Both self‐reported symptoms and clinical signs of TMD pain and dysfunction were significantly correlated with reduced OHRQoL as assessed by the OHIP14 questionnaire in the present study. Although clinical signs of pain and dysfunction were more prevalent than self‐reported symptoms, the latter were more strongly correlated with OHIP14 score, especially the self‐reported pain. Our results correlate well with findings from a systematic review that assessed the association between TMD and OHRQoL (Dahlstrom & Carlsson, 2010). This review found that TMD had a significant negative impact on OHRQoL, with the largest impact associated with self‐reported symptoms. This suggests that some of the clinically registered signs, especially of dysfunction, may be of little significance to the patients. To assess the burden of disease and for planning and evaluating interventions of TMD, the self‐reported measures of TMD symptoms may therefore be more relevant than the clinical signs.

There are numerous diagnostic systems in use for TMD associated pain and symptoms, some designed primarily for research, others for use in clinical practice (Ohrbach & Dworkin, 2016). In 2014 the Diagnostic criteria for TMD (DC/TMD) were published, which are common guidelines for diagnosing TMD in research and clinical practice, developed through workshops and validation projects (Ohrbach & Dworkin, 2016). The DC/TMD is now the dominant diagnostic system for TMD. The TOHNN study was performed before the DC/TMD was published, and the TMD examinations were done according to a different, previously described protocol (Yekkalam & Wanman, 2014a; Yekkalam & Wanman, 2014b). A recent study compared the diagnostic outcome of questions very similar to those used in the present study and those obtained by using the DC/TMD diagnostic system. The question on pain in the temple, face, jaw, or jaw joint pain once a week or more showed substantial validity in relation to TMD pain among adults, whereas the question related to frequent impairment of jaw functions showed fair to moderate validity to DC/TMD dysfunction (Lovgren, Visscher, et al., 2016). Comparison of TMD data obtained by different diagnostic criteria should be done with caution. However, TMD prevalence data seem to be surprisingly consistent across populations and diagnostic criteria, suggesting that the outcome is fairly reproducible between diagnostic systems (LeResche, 1997). In published literature, TMD prevalence data are often based on self‐reported pain in the jaw‐face area, with prevalence between 6–15% for women and 3–10% for men (LeResche, 1997). The corresponding prevalence of self‐reported jaw‐face pain for both men and women in the present study were 2.0% and 5.6%, respectively, which are slightly lower than these previous TMD estimates.

Our study is the first to report the prevalence of symptoms related to TMD in a Norwegian adult population, and our results correlates well with reports from cross‐sectional studies of other populations. Our study is also one of few to report the correlation between symptoms of TMD and OHRQoL in a large general adult population, and our results shows that self‐reported symptoms of TMD had a stronger impact on OHRQoL than clinical signs.

## CONFLICT OF INTEREST

The authors declare that there is no conflict of interest.

## AUTHORS’ CONTRIBUTIONS

NO, BJ, and AT planned and design the study. NO, BJ, GEH organized the practicalities. NO, BJ, GEH, ET, and AT collected the data. AT, EHO, and ET performed literature research and analyzed the data. AT and ET drafted the manuscript. EHO and BJ were major contributors in writing the manuscript. All authors read and approved the final manuscript.

## Data Availability

'Data available on request due to privacy/ethical restrictions': The data that support the findings of this study are available on request from the corresponding author. The data are not publicly available due to privacy or ethical restrictions.
